# Supplementary material for: DeepEMhancer: a deep learning solution for cryo-EM volume post-processing
Source: Commun Biol. 2021 Jul 15;4:874. doi: 10.1038/s42003-021-02399-1 (PMC8282847; doi:10.1038/s42003-021-02399-1)
Supplement: Supplementary file 1 — Supplementary Material [file 42003_2021_2399_MOESM1_ESM.pdf]

DeepEMhancer: a deep learning solution for cryo-EM volume post-processing

Ruben Sanchez-Garcia, Josue Gomez-Blanco, Ana Cuervo, Jose Maria Carazo, Carlos Oscar S. Sorzano, Javier Vargas

Content

Supplementary Table 1: Neural network architecture 2

Supplementary Figure 1 4

Supplementary Note 1: Visual inspection of testing maps 5

Supplementary Figure 2 5

Supplementary Note 2: Map local quality impact on performance 6

Supplementary Figure 3 7

Supplementary Note 3: FSC curves of the studied maps 8

Supplementary Figures 4-9 9

Supplementary Note 4: Target selection impact in model performance 13

Supplementary Figure 10 13

Supplementary Figures 11-13 14

Supplementary Figures 14 and 15 17

Supplementary Note 6: Training targets generation workflow 19

Supplementary Figure 16 19

Supplementary Note 7: List of EMDB entries used in this work 20

Supplementary references 23

Supplementary Table 1. Neural network architecture

Layer	Type	Parents	#Kernels	Stride	Kernel size	Output shape
1	Conv3d+GN+PReLU	Input	32	1	5	64x64x64x32
2	Conv3d+GN+PReLU	1	32	1	5	64x64x64x32
3	Conv3d+GN+PReLU	2	32	2	5	32x32x32x32
4	Conv3d+GN+PReLU	3	64	1	5	32x32x32x64
5	Conv3d+GN+PReLU	4	64	1	5	32x32x32x64
6	Conv3d+GN+PReLU	5	64	2	5	16x16x16x64
7	Conv3d+GN+PReLU	6	128	1	5	16x16x16x128
8	Conv3d+GN+PReLU	7	128	1	5	16x16x16x128
9	Conv3d+GN+PReLU	8	128	2	5	8x8x8x128
10	Conv3d+GN+PReLU	9	128	1	5	8x8x8x128
11	Concat +Conv3d_trans	10	128	2	5	16x16x16x128
12	Conv3d+GN+PReLU	10 & 7	128	1	5	16x16x16x128
13	Conv3d+GN+PReLU	12	128	1	5	16x16x16x128
14	Conv3d+GN+PReLU	13	128	1	5	16x16x16x128
15	Concat +Conv3d_trans	14 & 4	64	2	5	32x32x32x64
16	Conv3d+GN+PReLU	15	64	1	5	32x32x32x64
17	Conv3d+GN+PReLU	16	64	1	5	32x32x32x64
18	Conv3d+GN+PReLU	17	64	1	5	32x32x32x64
19	Concat +Conv3d_trans	18 & 1	32	2	5	64x64x64x32
20	Conv3d+GN+PReLU	19	32	1	5	64x64x64x32
21	Conv3d+GN+PReLU	20	32	1	5	64x64x64x32
22	Conv3d+GN+PReLU	21	32	1	5	64x64x64x32

23	Conv3d_trans	22	16	2	5	128x128x128x16
24	Conv3d+GN+PReLU	23	8	2	5	64x64x64x8
25	Conv3d	24	1	1	5	64x64x64x1

Total number of parameters: 51,119,889

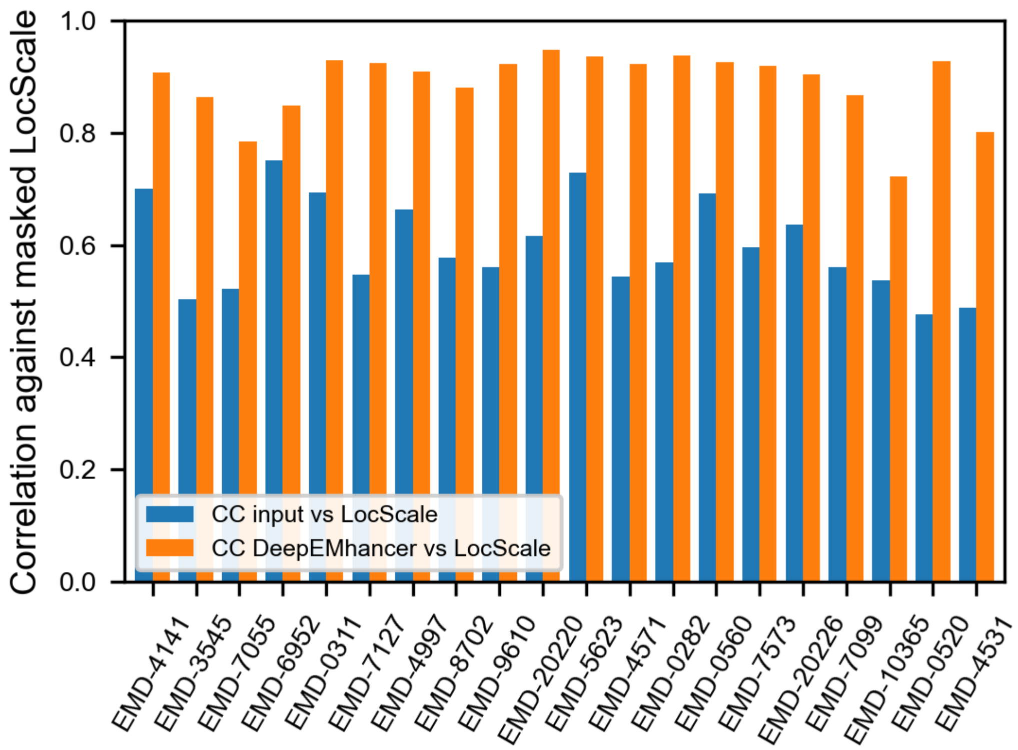

Supplementary Figure 1. Correlation coefficient for the input maps of the testing set before (blue) and after (orange) the treatment with DeepEMhancer compared against LocScale processed maps.

Supplementary Note 1: Visual inspection of testing maps

EMD-7055

The EMD-7055[1] is a medium resolution volume of the NAIP5-NLRC4-flagellin inflammasome. For the purposes of this article, the main interesting aspect of this volume is the apparent poor performance of DeepEMhancer according to Main Text Figure 1. One of the reasons behind this behaviour is the fact that a mask was applied to only three subunits at the last stages of the refinement process. As a result, the volume contains signal for both masked and unmasked subunits although their intensity levels vary severely. Consequently, our neural network has tried to restore both the originally masked and unmasked regions, and thus, the results are not as good as in the other cases. However, when the volume is carefully pre-processed in order to remove those unmasked subunits while preserving the normalization constraints, non-negligible improvements were observed. Secondly, another important reason for the poor measured metrics is the fact that the atomic model (PDB 6b5b) was obtained by means of rigid body fitting of an homology model instead of being traced, thus, the agreement between the atomic model and the density map is far from being perfect. As a result, the resolution estimates computed using the atomic model as reference are not too accurate.

Supplementary Figure 2 shows the overall aspect of the post-processed volume compared to the raw and the B-factor-sharpened ones. As can be appreciated in Supplementary Figure 2 panel A, the map produced by DeepEMhancer is much cleaner than the B-factor processed map. More importantly, although the level of detail in the core of the protein is similar, in the outer part of the protein, the B-factor sharpened map presents broken densities that look continuous in the map obtained with DeepEMhancer, thus facilitating the map interpretation.

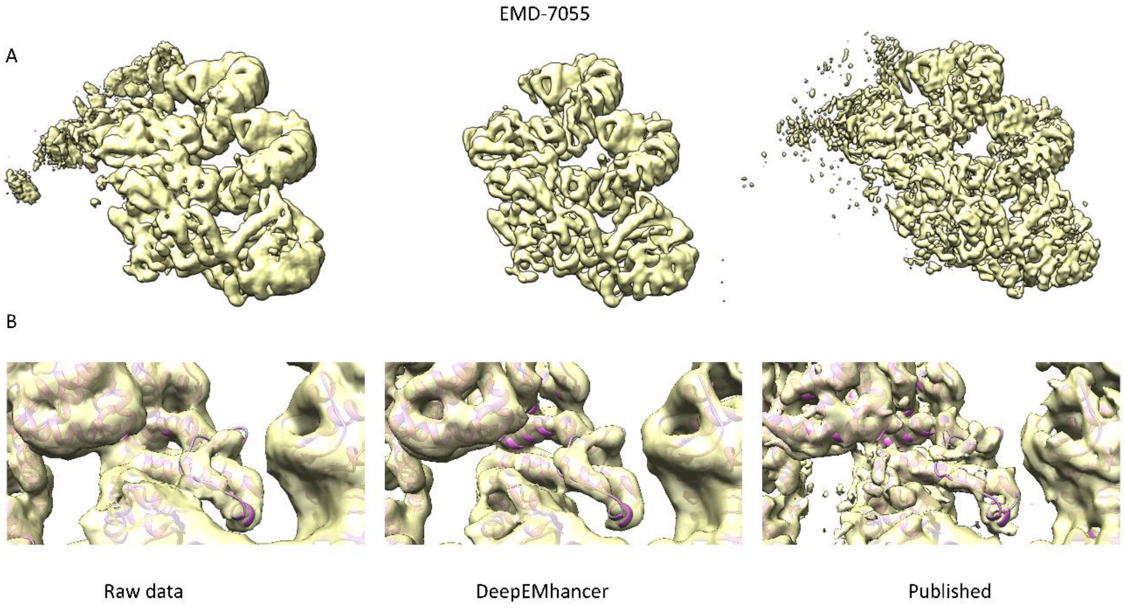

Supplementary Figure 2. DeepEMhancer post-processed volume for EMD-7055. A, overview of the raw data map, the post-processed map and the B-factor corrected map. B, zoom-in of a region containing the loop Q514-S533, which looks cleaner and better resolved in the DeepEMhancer post-processed map compared to the raw data and sharpened maps.

Supplementary Note 2: Map local quality impact on performance

In order to study how the local quality of the maps impacts the performance of DeepEMhancer, we have studied the local quality of the post-processed maps as a function of the local quality of the raw maps. Among all possible quality metrics that we could have computed, we used the local correlation between the post-processed map and the atomic model and between the raw map and the atomic model using a sliding window approach. Local resolution was discarded as a quality metric due to the wide variety of values found within the testing set. Local correlation was computed using a sliding window approach of 7x7x7 (or 5x5x5).

Supplementary Figure 3 displays the distribution of such correlation values for all maps included in the testing set. The maps were post-processed with LocScale (blue), DeepEMhancer (yellow) and with global B-factor correction as done in their publications (labelled as Published, red). As it is shown in Supplementary Figure 3, the three approaches tend to produce results of similar quality for high-quality input regions (correlation greater than 0.6). As expected, the quality of the post-processed regions decreases as the quality of the input data does, although such reduction is more pronounced for the global B-factor correction than for the other approaches, that seem comparable for the mid-quality range (correlation between 0.3 and 0.6). Finally, the quality of the post-processed maps is quite low for the correlation range between 0.1 and 0.3, being LocScale post-processed regions substantially better. In light of these results, we can state that the results produced by DeepEMhancer are more similar to the results produced by LocScale than the ones produced by global B-factor correction, and thus, they are better suited for maps of heterogeneous quality.

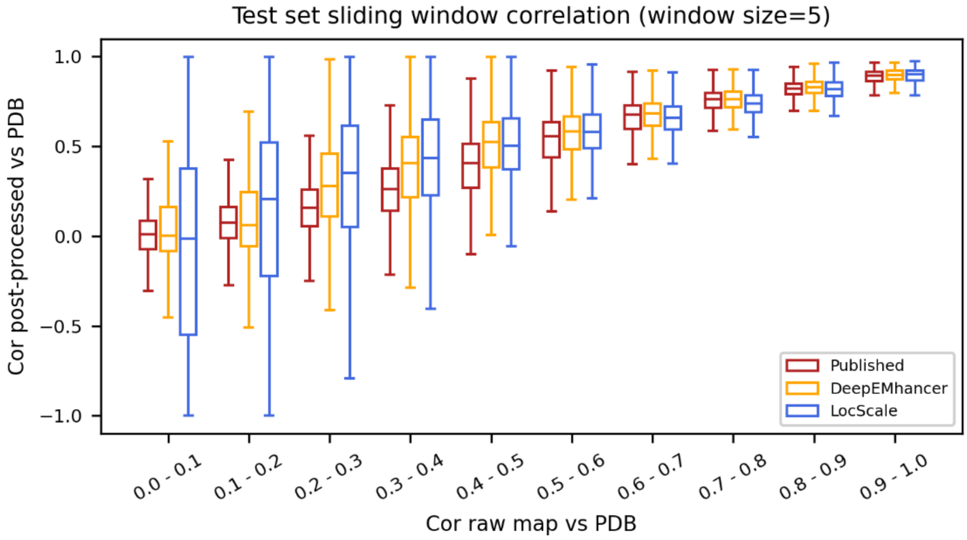

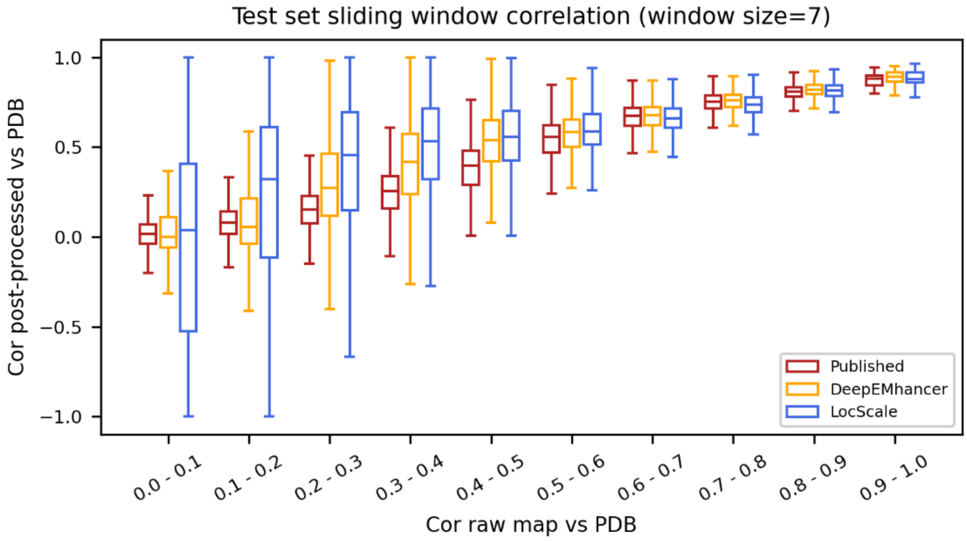

Supplementary Figure 3. Sliding window correlation between the post-processed maps and the reference (y-axis) and between the raw map and the reference (x-axis) using a window size of 5 (top) and 7 (bottom) voxels. Published maps were post-processed using global B-factor correction. Boxes enclose values between the first and third quartile (Q1 and Q3, lower and upper limit respectively). Median values are depicted as the horizontal lines within the boxes. Whiskers enclose values between Q1 - 1.5(Q3-Q1) and Q3 + 1.5(Q3-Q1).

Supplementary Note 3: FSC curves of the studied maps

This section includes the FSC curves of the post-processed maps computed against the reference map derived from the atomic model for the three maps that were presented in detail in the main text: EMD-7099, EMD-4997, and EMD-30178 (Supplementary Figures 4-6). Post-processed algorithms used were LocalDeblur, LocScale, AutoSharpen and DeepEMhancer. Please notice that LocalDeblur results are making use of the atomic model information for the masks calculation and that LocalScale makes use of such information in a direct manner. Consequently, their results are expected to be among the best. Nevertheless, DeepEMhancer tends to produce results that are similar to the training targets (LocScale-Masked), being of especial quality for the EMD-7099 and EMD-30178 cases.

One particular caveat that can seem confusing in our plots is the bounce that the curves of LocScale-Masked, DeepEMhancer and to a lesser degree, LocalDeblur experiment. Such bounces are caused by the tight mask employed being derived from the atomic model used as reference (see Supplementary Figure 7 and 8). Concisely, the fact that the maps obtained from the atomic models introduce high frequency components due to the nature of the atomic basis functions. Masking the maps equally introduces high frequency components due to the convolution of the Fourier transform of the map with the Fourier transform of the binary mask (see Supplementary Figure 9). As a consequence, the two maps present the same kind of behaviour at high frequency thus the correlation causing the bounces in the FSC. However, we want to highlight that those bounces, that occur at FSC values <0.3, are not relevant for the comparison against the atomic model, which should be done at threshold 0.5[4]. Nevertheless, if the bounces are desired to be removed, phase randomization could be applied to the post-processed map[5].

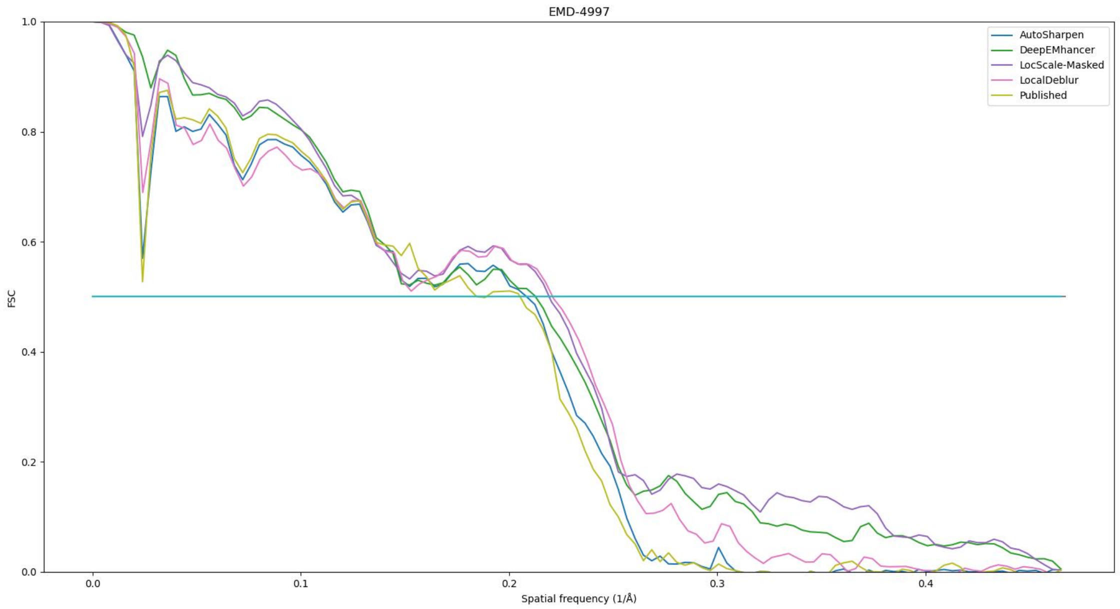

Supplementary Figure 4. FSC curves for the maps post-processed with AutoSharpen (blue), DeepEMhancer (green), masked LocScale (purple) and LocalDeblur (magenta) for the EMD-4997. FSC curves were computed using as reference the map derived from the atomic model.

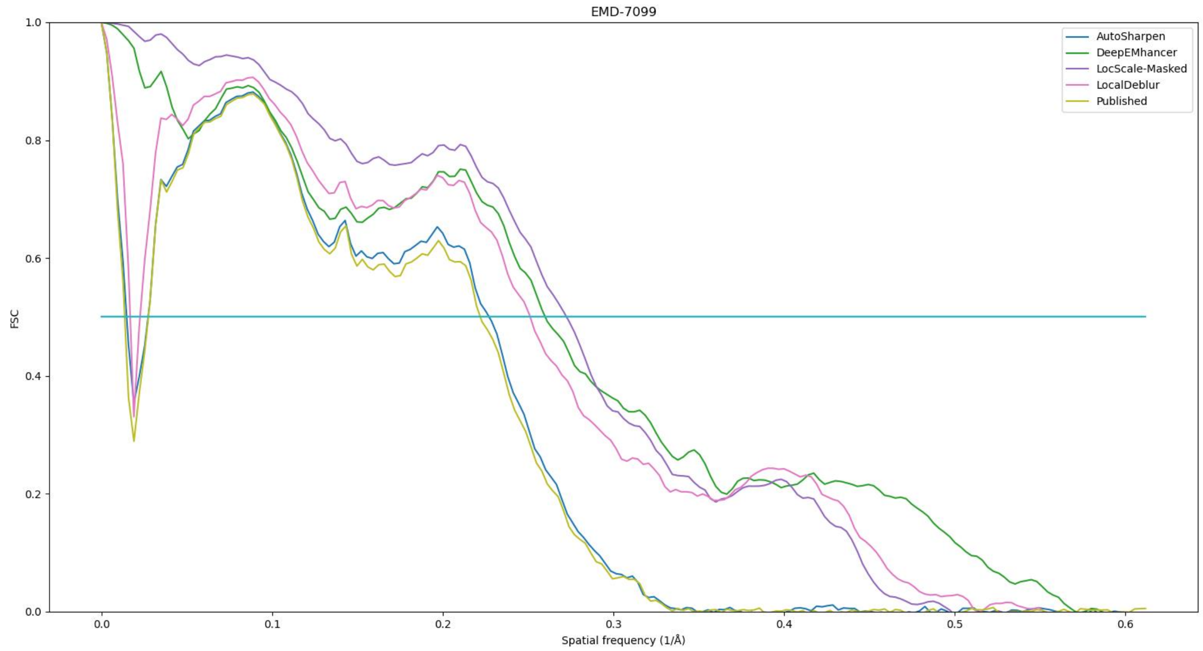

Supplementary Figure 5. FSC curves for the maps post-processed with AutoSharpen (blue), DeepEMhancer (green), masked LocScale (purple) and LocalDeblur (magenta) for the EMD-7099. FSC curves were computed using as reference the map derived from the atomic model.

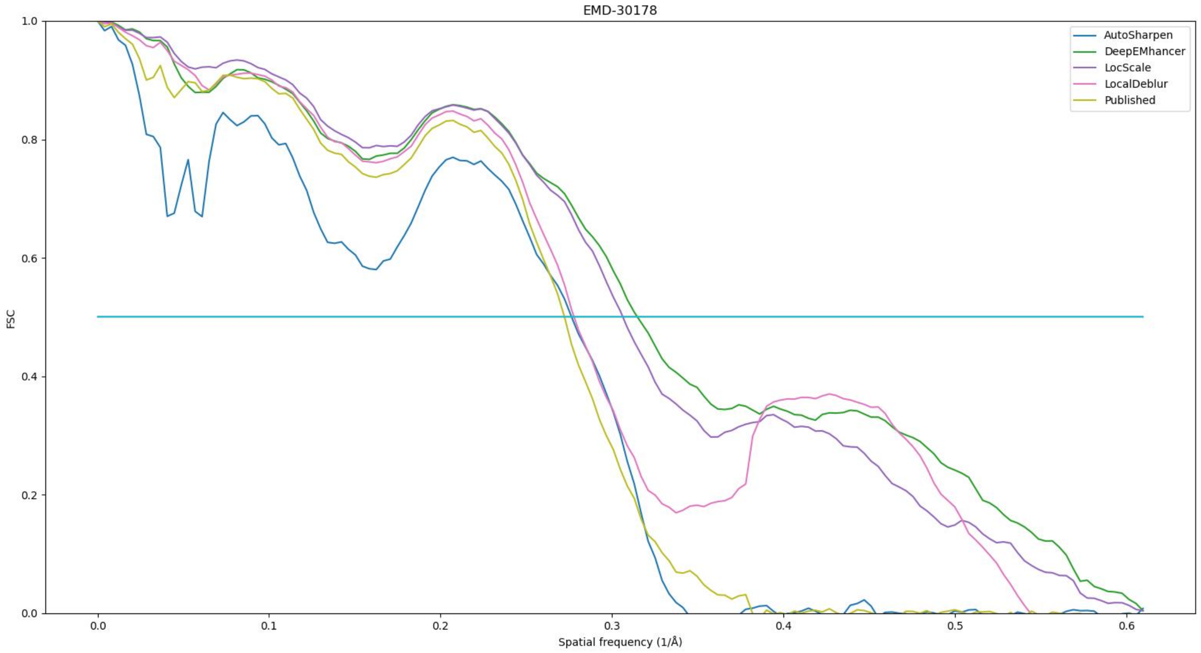

Supplementary Figure 6. FSC curves for the maps post-processed with AutoSharpen (blue), DeepEMhancer (green), masked LocScale (purple) and LocalDeblur (magenta) for the EMD-30178. FSC curves were computed using as reference the map derived from the atomic model.

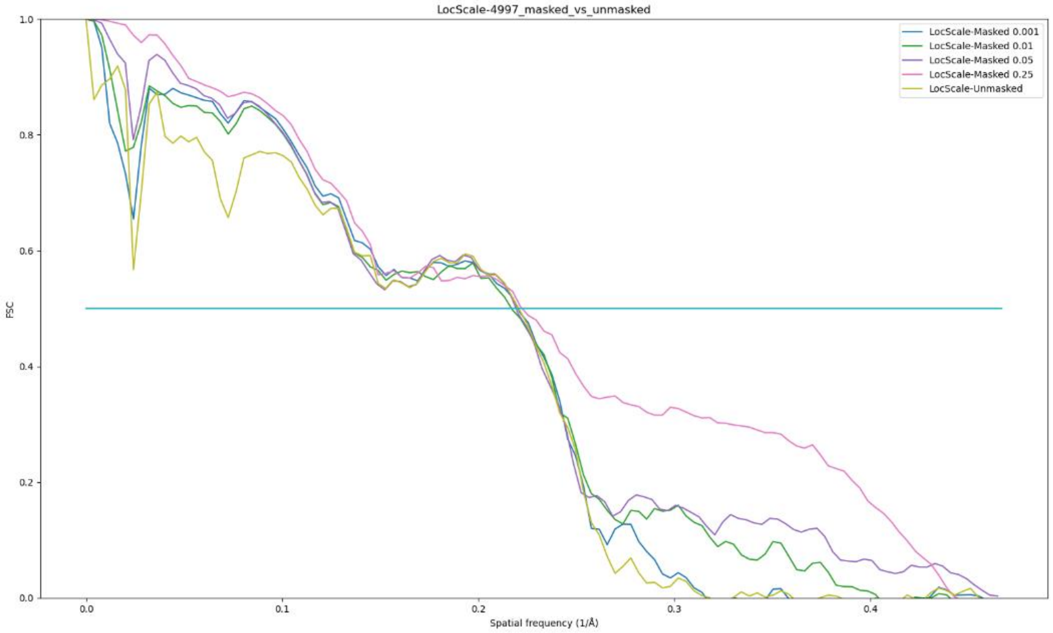

Supplementary Figure 7. FSC curves for the maps post-processed with LocScale for the EMD-4997 not using (golden) the training tight mask and using masks with different degrees of tightness (blue, green, purple and magenta sorted by increasing tightness). FSC curves were computed using as reference the map derived from the atomic model. The degree of tightness is measured as the relative threshold used for mask binarization (e.g., LocScale-Masked 0.01 is computed using the binary mask obtained using as threshold the percentile 1%) for the maps post-processed with LocScale for the EMD-4997 using (blue) and not using (green) the training tight mask. FSC curves were computed using as reference the map derived from the atomic model.

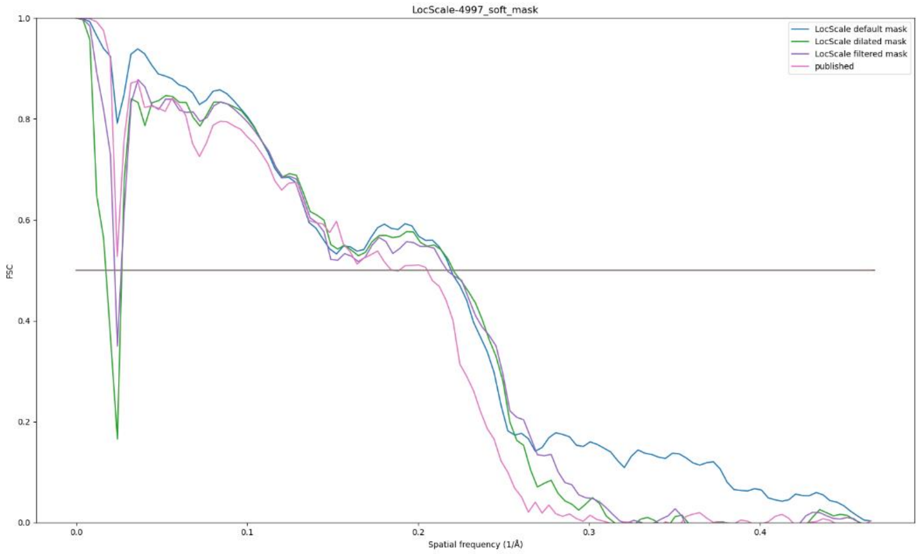

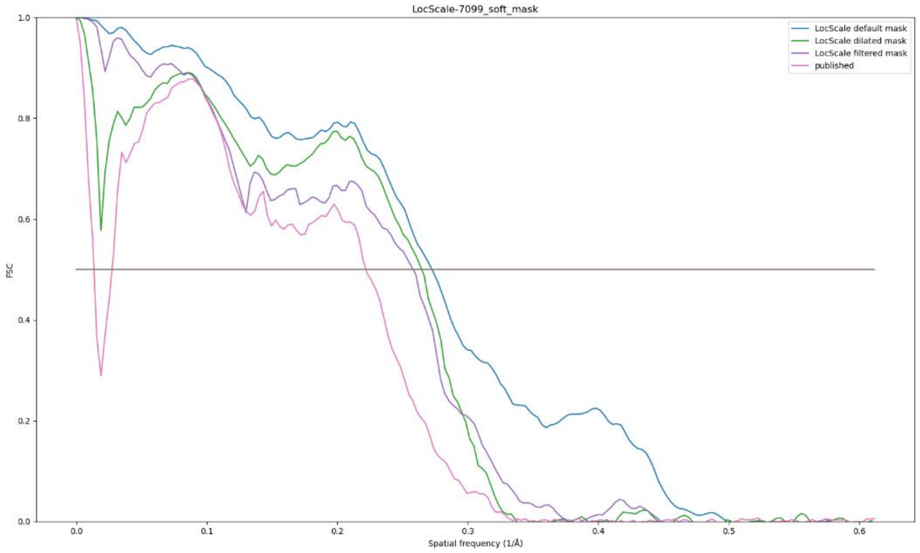

Supplementary Figure 8. FSC curves for the maps post-processed with LocScale for the EMD-4997 (top) and EMD-7099 (bottom) not using (magenta) the training tight mask and using the training mask (blue) and the train mask filtered (purple) or dilated (green). FSC curves were computed using as reference the map derived from the atomic model.

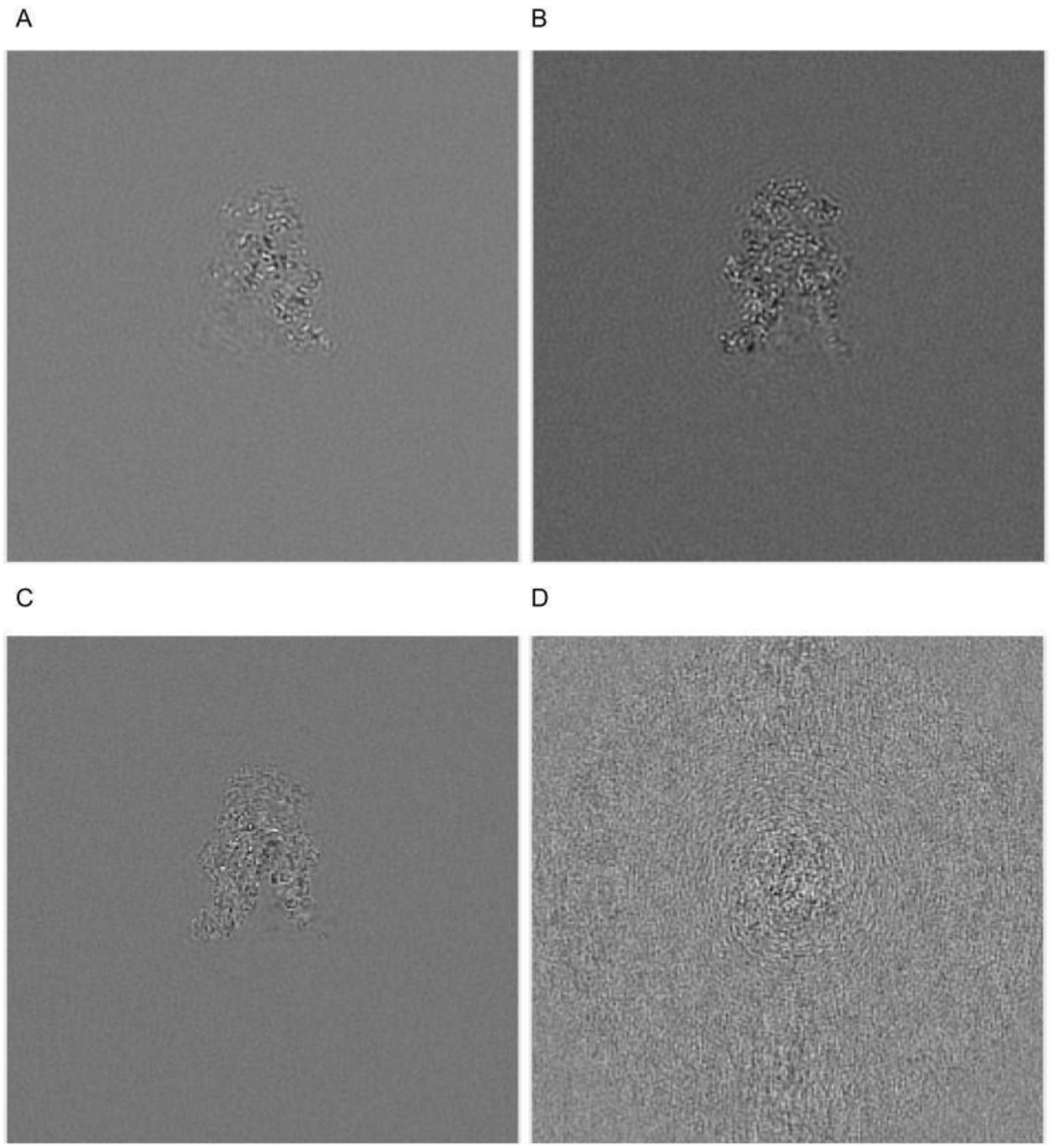

Supplementary Figure 9. Maps difference for EMD-4997 between DeepEMhancer and low-pass filtered DeepEMhancer (A), atomic model and filtered atomic model (B), LocScale-Masked and filtered LocScale Masked (C) and experimental map and filtered experimental map (D). Border induced artifacts can be observed in panels A-C.

Supplementary Note 4: Target selection impact in model performance

DeepEMhancer has been originally trained using as targets tightly masked volumes that were sharpened with LocScale. We also tried to train another version using as targets simulated maps derived directly from the atomic models. Although the latter option seemed to provide more accurate targets, what we found was that our implementation was not able to learn in detail how to reproduce such targets. As a consequence, the overall performance of DeepEMhancer trained using atomic models was inferior to the one trained on post-processed maps. Serve as examples the slices shown in Supplementary Figures 10-12 that illustrate how the results obtained with simulated targets look blurrier than the ones trained on post-processed maps both in training (C) and validation sets (A, B), which indicates severe underfitting.
Learning curves (Supplementary Figure 13) also indicate that DeepEMhancer was not able to accurately reproduce the atomic models targets since the loss function quickly plateaus after a small reduction. In light of these evidences, as we are using the same inputs and models and the only difference between the experiments is the selected targets, we have shown that, at least for our approach, learning to reproduce atomic model targets is more difficult than post-processed maps, leading to poorer results under our available computational resources and dataset.

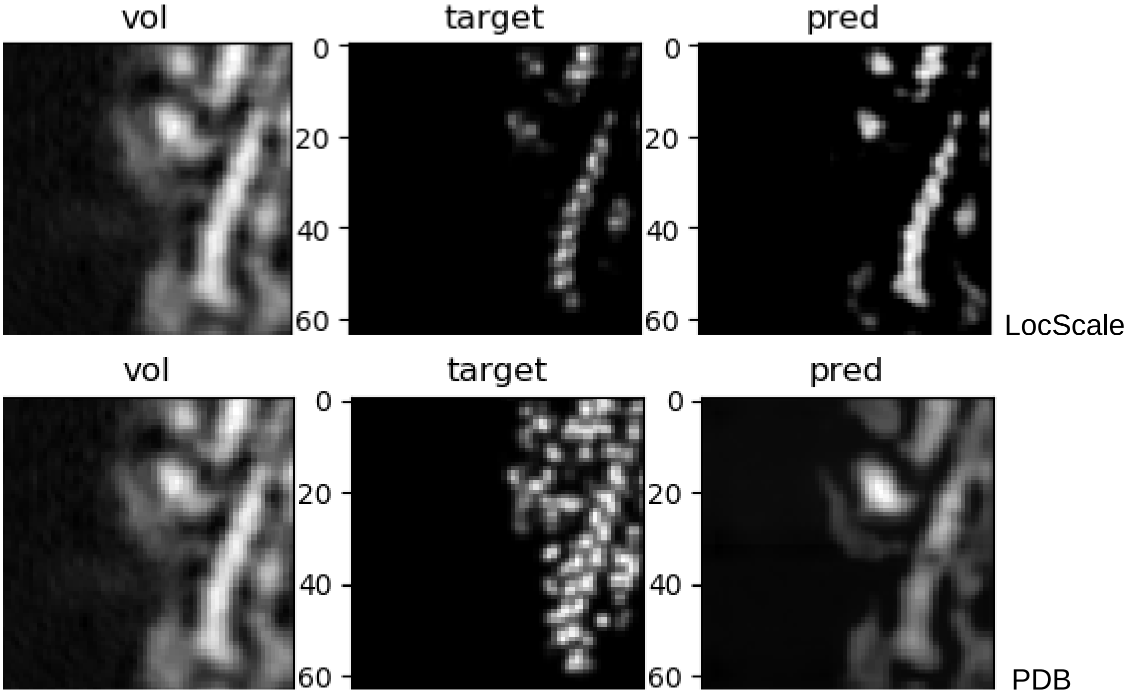

Supplementary Figure 10. Central slice of a 64x64x64 cube from the validation set entry EMD-6847 processed by DeepEMhancer when trained on masked LocScale targets (upper row) and simulated maps derived from PDB (lower row). vol: input volume; target: reference volume to be reproduced; pred: volume produced by DeepEMhancer.

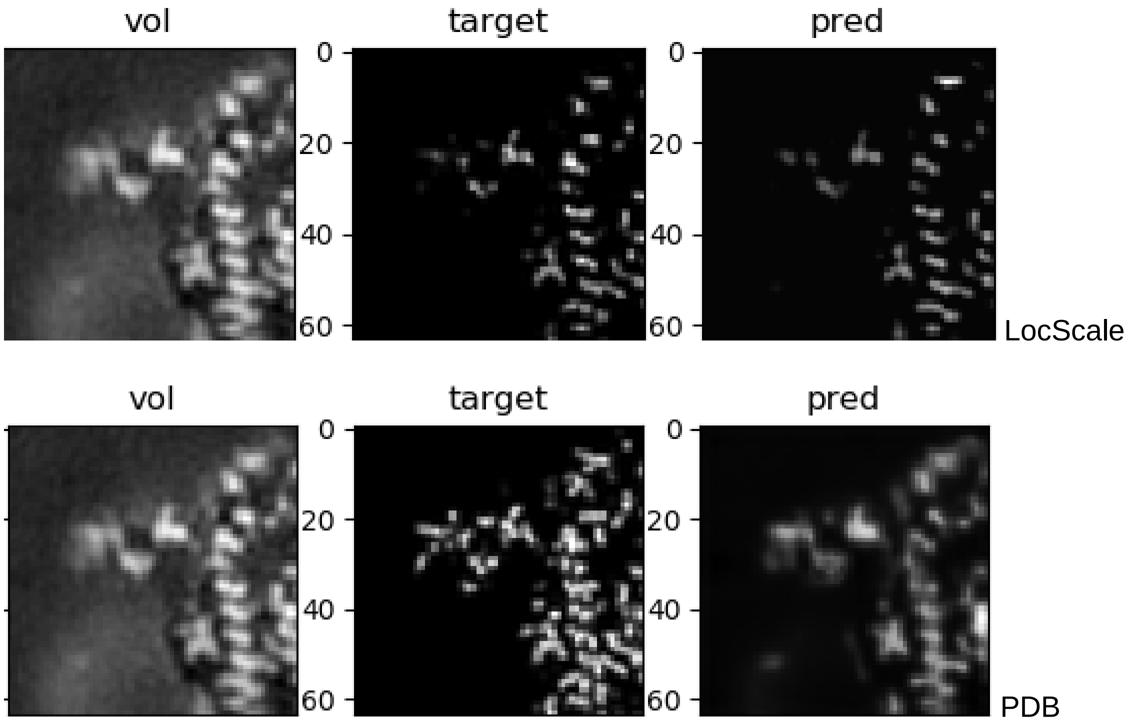

Supplementary Figure 11. Central slice of a 64x64x64 cube from the validation set entry EMD-9112 processed by DeepEMhancer when trained on masked LocScale targets (upper row) and simulated maps derived from PDB (lower row). vol: input volume; target: reference volume to be reproduced; pred: volume produced by DeepEMhancer.

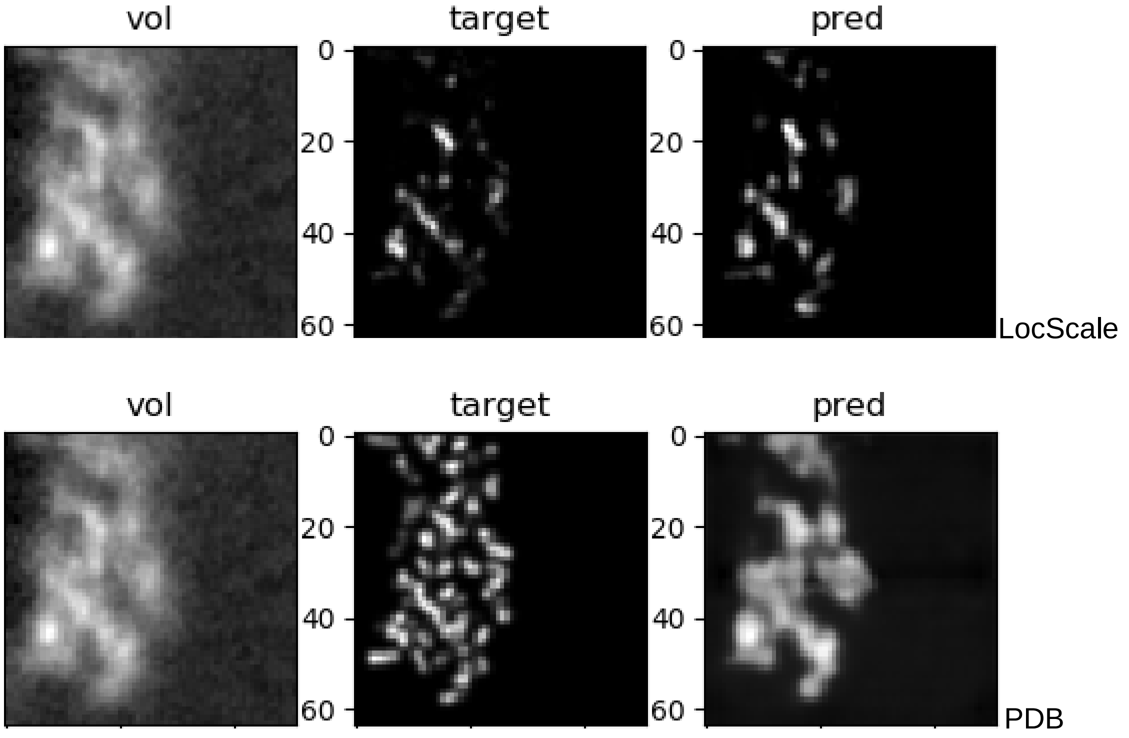

Supplementary Figure 12. Central slice of a 64x64x64 cube from the training set entry EMD-20986 processed by DeepEMhancer when trained on masked LocScale targets

(upper row) and simulated maps derived from PDB (lower row). vol: input volume; target: reference volume to be reproduced; pred: volume produced by DeepEMhancer.

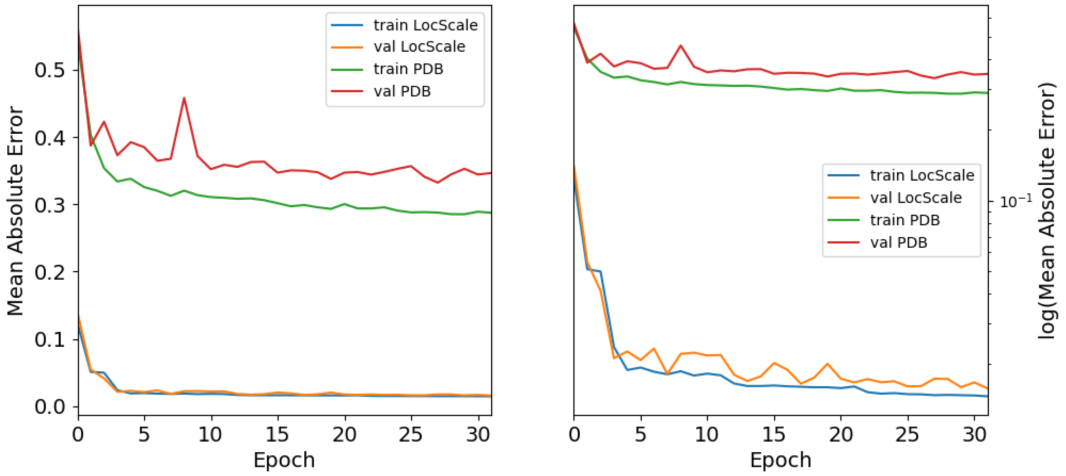

Supplementary Figure 13. Learning curves for DeepEMhancer using as targets masked LocScale post-processed volumes (blue and orange) and simulated from atomic models volumes (red and green). Both subplots differ only on the scale of the y-axis.

Supplementary Note 5: Dealing with post-translational modifications

Cryo-EM maps tend to exhibit heterogeneous local quality, leading to poorly defined regions in many macromolecules and consequently, unsolved regions in atomic models. This is especially true for the post-translational modifications that many residues may exhibit. Consequently, most atomic models do not include them (or only partially). Since we are making use of atomic models in the learning process, it is expected that our method will not deal well with such modifications. Indeed, we have realized that apart from a few glycans, little other examples were present in the training set. As a consequence, we have recorded a few successful examples in which glycans become more interpretable after DeepEMhancer. For instance, Supplementary Figure 14 shows one of such examples belonging to EMD-0282. Another successful instance can be found in Melero et al[6].

On the contrary when applied to other types of modifications, worse results are expected. However, since the training set contained also ligands, which are more diverse than residues, we expect that the network will not mask out the densities corresponding to the modifications providing their intensity is strong enough. Thus, serve as an example the EMD-9374 that contains the modified residue CRO ({2-[(1R,2R)-1-amino-2-hydroxypropyl]-4-(4-hydroxybenzylidene)-5-oxo-4,5-dihydro-1H-imidazol-1-yl}acetic acid). As displayed in the Supplementary Figure 15, the density corresponding to this modified residue looks shorter in the DeepEMhancer map, and although it was not totally masked out, it is better represented in the original map. Despite this map following the trend we expected, we cannot ensure that it will be the case for any possible map and as a consequence, we recommend the users to proceed with caution in the regions they expect to find post-translational modification.

Hopefully, this limitation will be reduced version after version of the program as the number of atomic models including post-translational modifications will increase in the future, when we will be able to retrain our model in a more representative dataset.

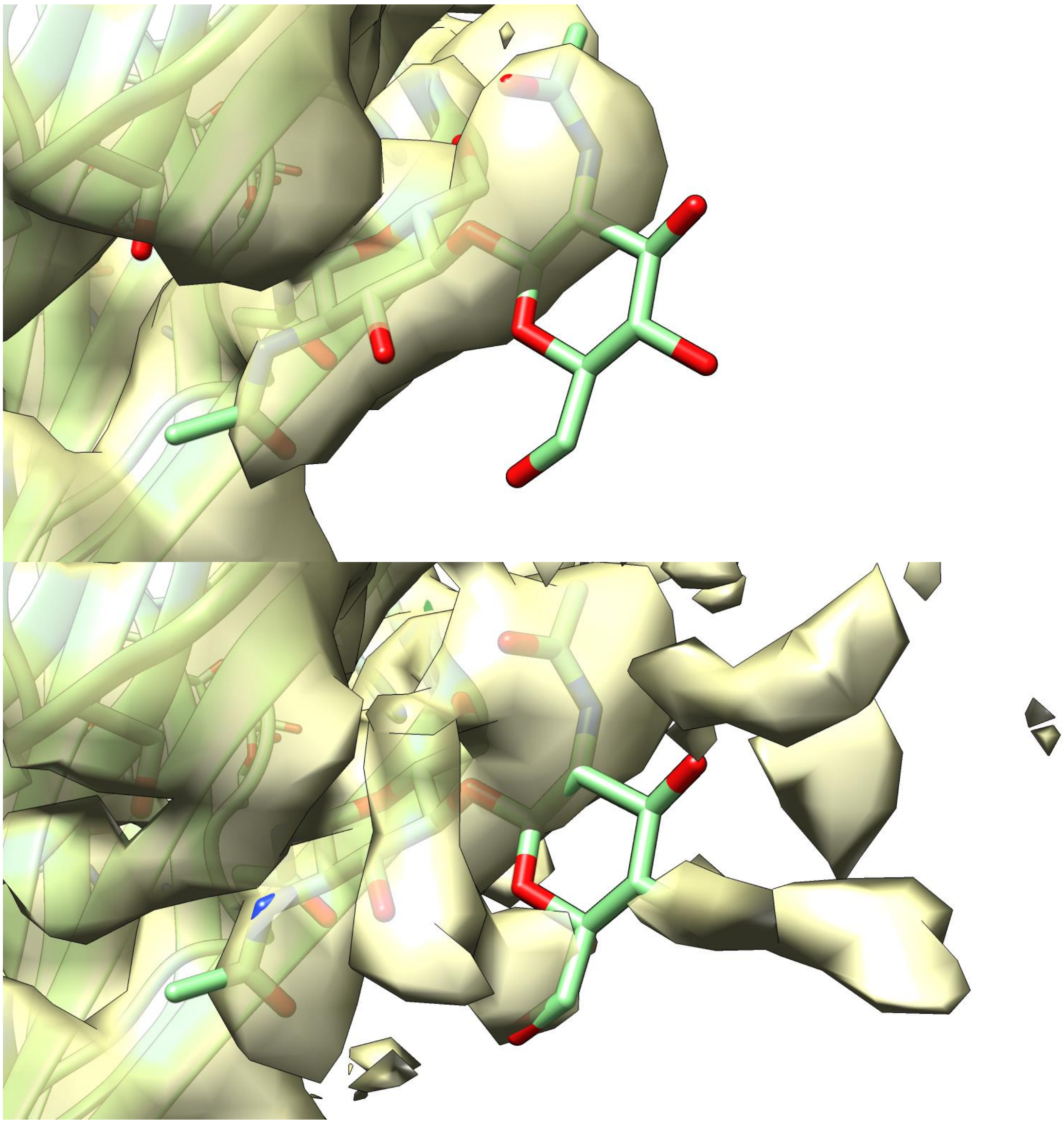

Supplementary Figure 14. Published map (bottom) and DeepEMhancer map (top) for the EMD-0282 centred at one NAG glycan.

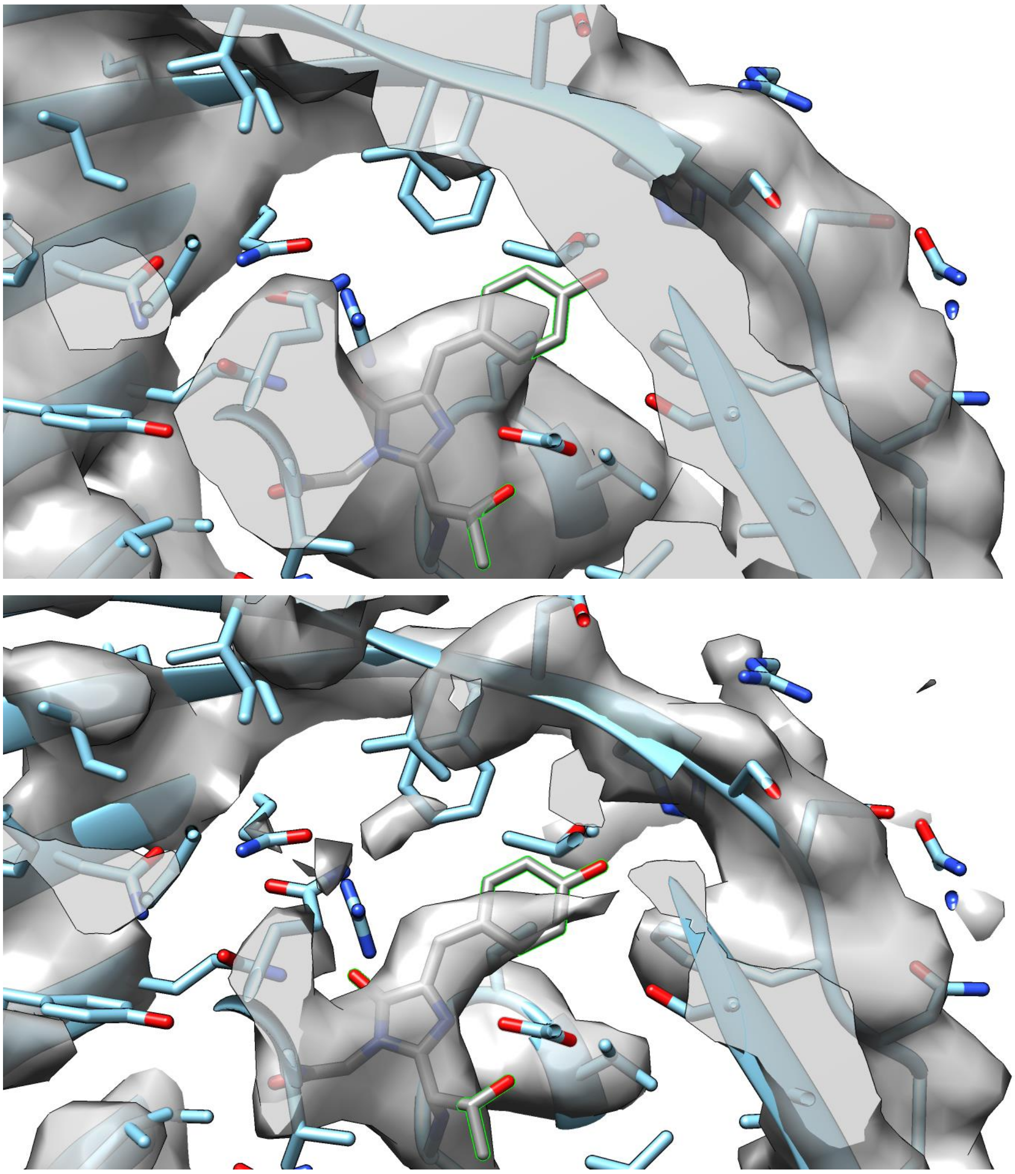

Supplementary Figure 15. Published map (bottom) and DeepEMhancer map (top) for the EMD-9374 centered at the CRO modified residue.

Supplementary Note 6: Training targets generation workflow

Atomic models associated with each EMD entry were downloaded from the PDB database. Then, as required by LocScale[2], we ruled out all entries which contained non-refined atomic B-factors. For each atomic model that survived atomic B-factor filtering, we computed continuous masks normalizing the simulated volumes that were produced with the e2pdb2mrc program from the EMAN-2 suite[3] using default parameters and the reported resolution. Percentile 95 was selected as threshold in order to obtain binary masks. Such tight masks were required during training in order to improve convergence since unmasked targets (that would require the network to learn how to predict random noise in order to get 0 loss) were producing worse results. Atomic models were also supplied as input, together with the average map computed from the half-maps, to the prepare_locscale_input.py program (provided in LocScale repository), that generated the corrected and reference volume required for LocScale execution. LocScale was run using as window size the recommended value of 7 * average_map_resolution / pixel_size. Finally, the computed output was masked using the aforementioned mask. As a result, we obtained as training targets LocScale post-processed and masked versions of the input volumes.

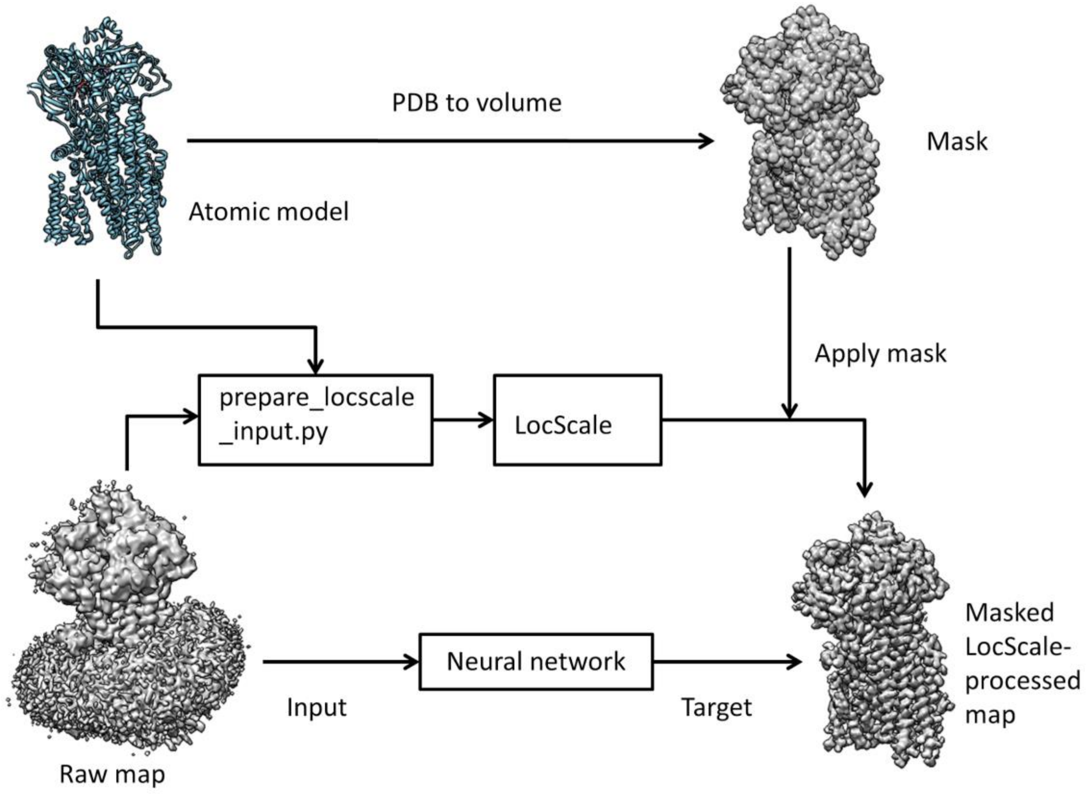

Supplementary Figure 16. Workflow employed to generate training data for DeepEMhancer neural network.

Supplementary Note 7: List of EMDB entries used in this work

Train

EMD-0026
EMD-0038
EMD-0071
EMD-0093
EMD-0094
EMD-0132
EMD-0234
EMD-0244
EMD-0408
EMD-0415
EMD-4288
EMD-0452
EMD-0490
EMD-0500
EMD-0501
EMD-0552
EMD-0567
EMD-0589
EMD-0592
EMD-0665
EMD-0776
EMD-10049
EMD-10069
EMD-10100
EMD-10105
EMD-10106
EMD-10134
EMD-10273
EMD-10279
EMD-10324
EMD-10333
EMD-10418
EMD-10534
EMD-10585
EMD-10595
EMD-10617
EMD-20145
EMD-20146
EMD-20189
EMD-20234
EMD-20249
EMD-20254
EMD-20259
EMD-20270
EMD-20271
EMD-20352
EMD-20521
EMD-20986
EMD-21012

EMD-21107
EMD-21144
EMD-21391
EMD-3661
EMD-3662
EMD-3802
EMD-3885
EMD-3908
EMD-4032
EMD-4073
EMD-4148
EMD-4162
EMD-4192
EMD-4214
EMD-4241
EMD-4272
EMD-4401
EMD-4404
EMD-4429
EMD-4588
EMD-4589
EMD-4593
EMD-4728
EMD-4746
EMD-4748
EMD-4759
EMD-4888
EMD-4889
EMD-4890
EMD-4907
EMD-4917
EMD-4918
EMD-4941
EMD-4983
EMD-6479
EMD-7009
EMD-7041
EMD-7065
EMD-7090
EMD-7334
EMD-7335
EMD-7770
EMD-7869
EMD-8437
EMD-8438
EMD-8911
EMD-8958
EMD-8960
EMD-9111
EMD-9258
EMD-9259
EMD-9891
EMD-9931
EMD-9934
EMD-9935

EMD-9939
EMD-9941
EMD-9695

Validation

EMD-0193
EMD-0257
EMD-0264
EMD-0499
EMD-10401
EMD-20133
EMD-20449
EMD-20508
EMD-20849
EMD-4611
EMD-4646
EMD-4733
EMD-4789
EMD-6847
EMD-7133
EMD-7882
EMD-8069
EMD-9112
EMD-9298
EMD-9374
EMD-9664

Test

EMD-0282
EMD-0311
EMD-0520
EMD-0560
EMD-10365
EMD-20220
EMD-20226
EMD-3545
EMD-4141
EMD-4531
EMD-4571
EMD-4997
EMD-5623
EMD-6952
EMD-7055
EMD-7099
EMD-7127
EMD-7573
EMD-8702
EMD-9610

Supplementary references

1. Tenthorey, J. L. *et al.* The structural basis of flagellin detection by NAIP5: A strategy to limit pathogen immune evasion. *Science (80-.).* **358**, 888–893 (2017).
2. Jakobi, A. J., Wilmanns, M. & Sachse, C. Model-based local density sharpening of cryo-EM maps. *Elife* **6**, (2017).
3. Tang, G. *et al.* EMAN2: An extensible image processing suite for electron microscopy. *J. Struct. Biol.* **157**, 38–46 (2007).
4. Rosenthal, P. B. & Henderson, R. Optimal determination of particle orientation, absolute hand, and contrast loss in single-particle electron cryomicroscopy. *J. Mol. Biol.* **333**, 721–745 (2003).
5. Chen, S. *et al.* High-resolution noise substitution to measure overfitting and validate resolution in 3D structure determination by single particle electron cryomicroscopy. *Ultramicroscopy* **135**, 24–35 (2013).
6. Melero, R. *et al.* Continuous flexibility analysis of SARS-CoV-2 spike prefusion structures. *IUCrJ* **7**, 1059–1069 (2020).
