# Supplementary material for: DeepEMhancer: a deep learning solution for cryo-EM volume post-processing
Source: Commun Biol. 2021 Jul 15;4:874. doi: 10.1038/s42003-021-02399-1 (PMC8282847; doi:10.1038/s42003-021-02399-1)
Supplement: Supplementary file 2 — Description of Supplementary Files [file 42003_2021_2399_MOESM2_ESM.pdf]

Description of Additional Supplementary Files

File name: Supplementary Data 1

Description: EMDB ids used in this work and cross-validation splits.

File name: Supplementary Data 2

Description: Data used to generate Figures 1-3 and Supplementary Figure 1. Rows represent measurements for each EMDB id. The header indicates to which map type each column refers.

File name: Supplementary Data 3

Description: Data used to generate Supplementary Figure 3. Two files, one for each window size was included. Each file contains the correlation coefficient measurement for each of the cube pairs for each of the studied methods.
